# Increased trunk movements in people with hereditary spastic paraplegia: do these involve balance correcting strategies?

**DOI:** 10.1007/s00415-022-11054-6

**Published:** 2022-03-20

**Authors:** Lotte van de Venis, Vivian Weerdesteyn, Aletta Konijnenburg, Bart P. C. van de Warrenburg, Alexander C. H. Geurts, Jorik Nonnekes

**Affiliations:** 1 Department of Rehabilitation, Center of Expertise for Parkinson and Movement Disorders, Donders Institute for Brain, Cognition and Behaviour, Radboud University Medical Center, PO Box 9101, 6500 HB Nijmegen, The Netherlands; 2 Department of Neurology, Center of Expertise for Parkinson and Movement Disorders, Donders Institute for Brain, Cognition and Behaviour, Radboud University Medical Center, Nijmegen, The Netherlands; 3 Department of Rehabilitation, Sint Maartenskliniek, Nijmegen, The Netherlands

**Keywords:** Hereditary spastic paraplegia, HSP, Balance, Gait, Postural control

## Abstract

**Objective:**

Hereditary spastic paraplegia (HSP) is characterized by a bilaterally spastic gait pattern. During gait, increased trunk movements are often observed. People with HSP likely generate trunk movements to improve foot clearance and step length, but there may be additional explanations. Here, we investigate whether there is an association between reduced balance performance and increased trunk movements, as an increase in trunk movements may partly reflect balance correcting strategies.

**Methods:**

We analyzed an historic cohort of 86 people with HSP who underwent gait analysis and balance examination. Two researchers reviewed gait analyses videos and classified the observed trunk movement as (1) normal, (2) moderately increased, or (3) markedly increased, and categorized participants as ‘toe walkers’ (yes/no). Balance performance and spatiotemporal gait parameters were collected from the medical files. Parameters were compared between people with normal vs. moderately increased trunk movements, moderately vs. markedly increased trunk movements, and normal vs. markedly increased trunk movements.

**Results:**

Patients with moderately increased trunk movements during gait scored lower on the Berg Balance Scale (*p* = 0.002) and/or the Mini Balance Evaluation Test (*p* = 0.043) than patients with normal trunk movements. Likewise, patients with markedly increased trunk movements performed worse on the BBS (*p* = 0.037) and/or the Mini-BESTest (*p* = 0.004) than patients with moderately increased trunk movements. Patients with markedly increased trunk movements were more often toe walkers than patients with moderately increased (68% vs. 6%; *p* < 0.001).

**Conclusions:**

We found an association between increased trunk movements and reduced balance capacity. This may have several—not mutually exclusive—explanations. One of these explanations is that trunk movements, at least partly, reflect balance correcting strategies. With the disease progression, ankle strategies and foot placement strategies become impaired and insufficient to restore balance after intrinsic perturbations. Hip strategies are then potentially recruited to maintain balance, resulting in increased trunk movements.

## Introduction

Hereditary spastic paraplegia (HSP) is a heterogeneous group of neurodegenerative disorders. Pure forms are clinically characterized by bilateral progressive spasticity. With the disease progression, people with HSP often develop muscle weakness and contractures in the lower extremities (e.g., a pes equinovarus deformity) [1–3]. Together with impaired proprioception, these motor impairments often lead to reduced balance and gait capacities, which are among the most disabling consequences of HSP [4, 5]. In complex forms, additional neurological symptoms are present, such as ataxia, mental retardation, peripheral neuropathy and/or optic atrophy [1, 2]. As the disease progresses, balance and gait problems become gradually more severe, and people with HSP typically develop a bilaterally spastic gait pattern [5, 6]. Increased truncal movements during gait in frontal, sagittal and transversal planes are reported from the early phases of the disease [7–9]. In daily clinical practice, the most pronounced trunk movements seem to be present in relatively young patients, and in patients without plantigrade foot contact during gait (‘toe walkers’). Muscle weakness and spasticity are rarely found in the trunk of people with HSP. Therefore, increased trunk movements during gait likely reflect a secondary phenomenon or compensation strategy [8, 10].

The clinical determinants underlying increased trunk movements during gait in people with HSP have rarely been studied. Assumingly, trunk movements are partly made in a compensatory effort to improve foot clearance and step length, but there may be additional explanations [8, 11, 12]. Here, we explore the hypothesis that increased trunk movements during gait in people with HSP partly reflect balance correcting strategies, specifically, the recruitment of so-called hip strategies. Hip strategies consist of the rotation of upper body segments around the center of mass, for instance, by making trunk or arm movements, and are usually recruited when other balance strategies are unavailable or insufficient [13, 14]. To investigate this hypothesis, we assessed whether the magnitude of truncal movements during gait in people with HSP was associated with a reduced clinical balance capacity. In addition, we explored whether increased trunk movements coincided with a higher percentage of toe walking.

## Methods

The Center of Expertise for Rare and Genetic Movement Disorders at Radboudumc Nijmegen, part of the European Reference Network for Rare Neurological Diseases (ERN-RND), is a primary national referral center for patients with HSP. In our expertise center, the diagnosis of HSP is based on clinical physical examination combined with clinical inheritance pattern and/or proven molecular defect. In the presence of disabling gait impairments, 3D-instrumented gait analysis is performed in our movement laboratory. For the current study, an historic cohort of patients was included, based on the following inclusion criteria: (1) established diagnosis of HSP, (2) the availability of a gait analysis performed between October 2013 and May 2021, and (3) the availability of documented balance scores [Berg Balance Scale (BBS) and/or Mini Balance Evaluation System Test (Mini-BESTest)]. As some participants performed multiple gait analyses during the selected time period, the first gait analysis with a documented balance score was included for analysis. If both Berg Balance Scale and Mini-BESTest scores were available, both scores were included for analysis. Exclusion criteria consisted of concomitant neurological or orthopedic conditions, inability to walk eight meters barefoot without walking aids, and age below 18 years. Prior to the gait analysis, 16 retroreflective markers were placed on the lower extremities according to the standard Plug-In Gait marker model for lower body. Patients then walked barefoot over an eight-meter walkway at a self-selected speed. The gait pattern was recorded with an eight-camera motion analysis system (VICON, Oxford UK) and two video cameras, one capturing the frontal plane and the second the sagittal plane. Two researchers (LV, JN) reviewed videos from the gait analyses in both the frontal (i.e., trunk lateroflexion) and sagittal plane (i.e. trunk flexion and extension) and classified the observed trunk movement as (1) normal, (2) moderately increased, or (3) markedly increased. In addition, patients who walked without plantigrade (heel touching the floor) contact during the stance phase were classified as ‘toe walkers’. Cohen’s kappa coefficients were calculated to determine the inter-rater reliability between the two raters for the classification of trunk movements during gait. Any initial disagreements between the raters were discussed and resolved in consensus. Spatiotemporal gait parameters (walking velocity, cadence, step length, step width) were calculated based on marker data in Vicon Polygon. The data were checked for normality. Independent samples *t* tests or Mann–Whitney *U* tests were used where appropriate to assess differences between groups on balance scores and spatiotemporal parameters. Comparisons were made between persons with normal vs. moderately increased trunk movements, between persons with moderately vs. markedly increased trunk movements, and between persons with normal vs. markedly increased trunk movements. Differences in the percentage of toe walkers between the three categories were assessed using Chi-square tests.

## Results

In total, 147 persons diagnosed with HSP, and with available gait analyses and balance scores were screened for eligibility. Sixty participants were excluded for the following reasons: concomitant neurological or orthopedic conditions (*n* = 15), inability to walk eight meters barefoot without the use of walking aids (*n* = 38), or age below 18 (*n* = 8). As a result, 86 (58 men) participants were included with a mean age of 48 years (range 19–75 years). A molecular diagnosis was present in 43 participants: [SPG4 (*n* = 26); SPG7 (*n* = 5); SPG30 (*n* = 3), SPG31 (*n* = 3), SPG11 (*n* = 2), SPG10 (*n* = 1), SPG17 (*n* = 1), SPG3A (*n* = 1) and SPG9 (*n* = 1)].

The initial absolute interrater agreement in the classification of trunk movements during gait was 83% (*κ* = 0.74). After discussion and through consensus, 100% agreement was reached.

**Fig. 1:**
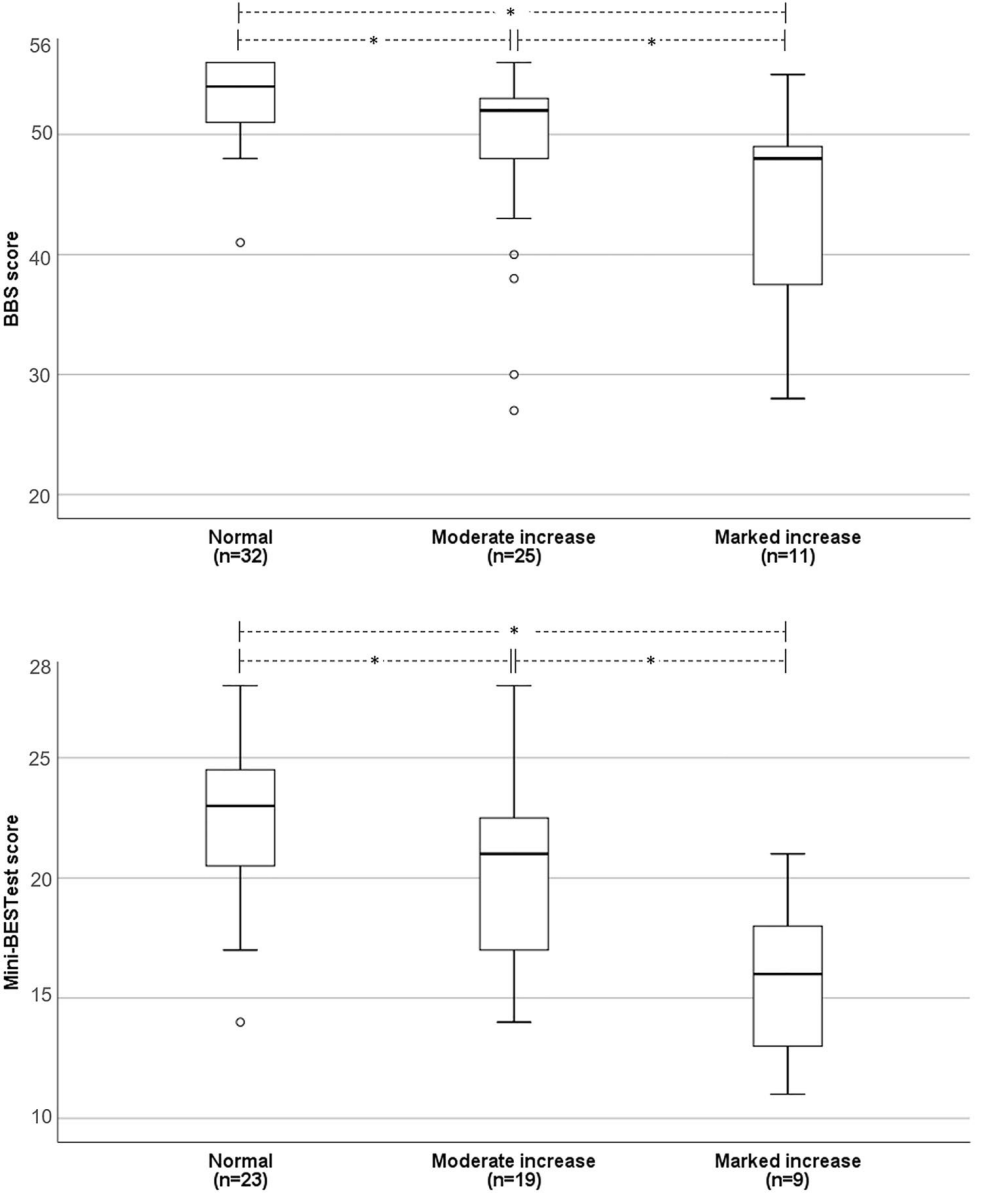
Balance scores for each level of trunk movements during gait in participants with HSP (*n* = 86). Median, interquartile range, and total range of balance scores (o indicating outliers) are shown per level of trunk movements. For a total of 33 participants both Mini-BEST test scores and BBS test scores were available. *BBS* Berg Balance Scale, *Mini-BESTest* Mini Balance Evaluation Test. *Significant differences between categories

Thirty-five participants (41%) were classified as having moderately increased trunk movements, while 13 participants (15%) had markedly increased trunk movements. Thirty-eight participants (44%) were classified as having normal trunk movements. Participants with markedly increased trunk movements were on average younger than participants with moderately increased trunk movements (33.2 ± 13 years vs. 48.2 ± 11 years; *p* < 0.001). Participants with normal trunk movements were 51.8 ± 12 years old, which was significantly different from the group with markedly increased trunk movements (*p* < 0.001), but not from the group with moderately increased trunk movements. Figure 1 shows the balance scores for each category of observed trunk movements for 68 participants (BBS score) and 51 participants (Mini-BESTest score); note that both balance scores were available for 33 participants. Participants with moderately increased trunk movements scored 2.0 points lower on the BBS (*p* = 0.002) and 2.3 points lower on the Mini-BESTest (*p* = 0.043) than those with normal trunk movements. Participants with markedly increased trunk movements scored 4.0 points lower on the BBS (*p* = 0.037) and 4.6 points lower on the Mini-BESTest (*p* = 0.004) than those with moderately increased trunk movements. Participants with markedly increased trunk movements scored 6.0 points lower on the BBS (*p* < 0.001) and 6.9 points on Mini-BESTest (*p* < 0.001) as compared to participants with normal trunk movements.

In total, 11 participants (13%) were categorized as toe walkers. They were on average younger than participants without toe walking (37.0 ± 14 vs. 49.1 ± 12 years; *p* = 0.003). The group of participants with markedly increased trunk movements included a higher percentage of toe walkers than the group with moderately increased trunk movements (69% vs 6%; *p* < 0.001). None of the patients with normal trunk movements showed toe walking.

Table 1 shows the spatiotemporal gait parameters for each level of observed trunk movements during gait. Participants with moderately increased trunk movements showed on average 0.07 m/s lower gait velocity (*p* < 0.022) and 0.05 m smaller step length as compared to those with normal trunk movements (*p* < 0.007). Participants with markedly increased trunk movements had 0.04 m smaller step width as compared to those with moderately increased trunk movements (*p* = 0.015). Participants with markedly increased trunk movements showed on average 0.06 m/s lower gait velocity (*p* = 0.030), 0.04 m smaller step width (*p* = 0.021), and 13 steps per minute lower cadence (*p* < 0.001), as compared to those with normal trunk movements. Other comparisons were nonsignificant.

**Table 1:** Spatiotemporal gait parameters for each level of trunk movements in participants with HSP

	Trunk movements during gait		
	Normal (*n* = 35)	Moderately increased (*n* = 38)	Markedly increased (*n* = 13)
Walking speed (m/s)	0.93 [0.72–1.35]	0.86 [0.26–1.26]*	0.87 [0.53–1.04]***
Cadence (steps/min)	103.8 ± 10.0	97.9 ± 15.6	90.8 ± 12.3***
Step length (m)	0.55 ± 0.07	0.50 ± 0.10*	0.53 ± 0.07
Step width (m)	0.19 [0.13–0.38]	0.19 [0.13–0.37]**	0.15 [0.09–0.42]***
Toe walkers (% of subgroup)	0%	6%**	69%

Values displayed are means (± standard deviation) or median [range]

*Significant differences between patients with normal trunk movements and moderately increased trunk movements

**Significant differences between patients with moderately increased trunk movements and markedly increased trunk movements

***Significant differences between patients with normal trunk movements and markedly increased trunk movements

## Discussion

In this historic cohort study, we investigated whether increased trunk movements during gait in people with HSP were associated with reduced balance capacity. Previous studies already reported increased trunk movements during gait in people with HSP in comparison to healthy controls [7–9, 15]. The current study has added value by exploring the potential association between increased trunk movements and clinical balance performance. Although our retrospective and cross-sectional study design does not allow inferences about causality, we found an association between increased trunk movements and reduced balance capacity. This association may have several—not mutually exclusive—explanations. One of these explanations is that trunk movements, at least partly, reflect balance correcting strategies.

Balance perturbations can be due to extrinsic factors (such as an icy pavement) or intrinsic factors (such as an impaired anticipatory postural adjustment to self-initiated movement). In people with HSP, intrinsic factors appear to play an important part. For instance, calf muscle spasticity may result in a sudden knee extension during the single-stance phase of gait, or cause retropulsion in a sit-to-stance transfer, jeopardizing postural stability [4]. Compensatory trunk movements for enlarging step length and foot clearance during gait [8, 11, 12] may also act as balance perturbations. Following an intrinsic balance perturbation, balance needs to be restored reactively. In general, humans have three strategies to restore balance during gait (1): the foot placement strategy, where people alter foot placement of the swing leg to adjust the base of support [13, 14, 16–18]; (2) the ankle strategy, where ankle moments of the stance leg are modulated to make (minor) adjustments to center-of-mass (CoM) movements [13, 16, 18, 19]; and (3) the hip strategy, where upper body segments are rotated around the CoM [13, 14]. When all three strategies are available for balance recovery, foot placement adjustment strategies and ankle strategies are preferred, while hip strategies are generally recruited when both foot placement and ankle strategies are insufficient; for instance, when walking on a narrow beam [14], when performing under time pressure (i.e., responding to a perturbation that occurs just prior to foot contact, leaving insufficient time to perform an adequate foot placement adjustment [20–22]), or when affected by a neurological disease [15].

People with HSP may be limited in using the ankle strategy for balance corrections due to lower-extremity spasticity (e.g., of the calf muscles), muscle weakness (e.g. of the ankle dorsiflexors), and/or ankle–foot deformities (e.g., pes equinus or pes equinovarus), or even sensory or cerebellar ataxia [4]. To compensate, patients become more dependent on the foot placement strategy [17]. When adjustments in the mediolateral direction are needed, for example, step width must be increased. In more severely affected individuals with HSP, adjustments in foot placement themselves may be impaired due to proximal lower-extremity spasticity (e.g. of the hip adductors), ataxia, and/or apparent slowness of postural responses [4]. In these patients, the recruitment of hip strategies may become particularly important to maintain balance during gait, which would explain the presently reported increased trunk movements. Although less likely, we cannot rule out truncal ataxia as an alternative explanation for increase trunk movements as we did not assess its presence at the time of the instrumented gait analysis.

In support of this line of reasoning, a previous study reported that after restoring the prerequisites for recruitment of ankle strategies and foot placement strategies in a patient with HSP and bilateral structural pes equinus (through bilateral Achilles tendon lengthening), a clear decrease in trunk movements was observed, suggesting less reliance on hip strategies to maintain balance during gait [15]. In addition, studies in other populations have demonstrated increased trunk movements when ankle strategy recruitment was artificially hampered. For example, in nine healthy participants, bilateral foot- and ankle-immobilizing casts were used to limit ankle strategy recruitment. During gait, trunk lateroflexion increased in the cast condition as compared to walking with lightweight sneakers [23]. Comparably, in children with cerebral palsy, rigid ankle–foot orthoses hampering the recruitment of ankle strategies were found to increase lateral trunk movements as compared to barefoot walking [24, 25] and as compared to walking with shoes without orthoses [26]. As plantigrade foot contact is a prerequisite for effective use of ankle strategies [4], the loss of ankle strategies likely explains the large proportion of toe walkers in our patient category with markedly increased trunk movements.

A disadvantage of increased trunk movements during walking is the inevitable rise in mechanical energy costs related to larger CoM excursions, which most likely results in greater metabolic costs as well. A commonly used method to assess the metabolic costs of walking is indirect calorimetry [27]. Regrettably, indirect calorimetry is not part of our routine clinical gait analysis and could therefore not be included in the current analyses. In line with our clinical observations, our results indicate that markedly increased trunk movements during gait were predominantly present in relatively young people with HSP. It may be that younger people with HSP are able to walk with markedly increased trunk movements, although energy demanding, whereas this is often too demanding for older people with HSP, who therefore opt for the use of walking aids. Alternatively, we found that the group of toe walkers was on average younger than those without toe walking. It might be that people with an earlier disease onset progressed further (resulting in more severe spasticity and development of contractures resulting in pes equinus), as compared to ambulatory older people with HSP. Both suggestions require further investigation.

This study has several limitations that should be taken into account. First, an objective assessment of trunk movements during gait was lacking, as 3D gait analyses were performed using the lower body marker model. A second limitation is the lack of other clinical participant characteristics, such as disease duration, degree of spasticity and muscle weakness of the lower extremities, presence of sensory impairments, or an indicator of disease severity (e.g., the Spastic Paraplegia Rating Scale) [28]. Hence, we could not test whether disease progression itself was associated with increased trunk movements. In addition, it was not possible to extract item subscores off e.g., the BBS from the medical files to investigate the notion that increased truncal movements while walking—although unlikely—might be associated with truncal instability or ataxia while sitting. Thirdly, we could not make use of the same balance score for all participants. This was due to the fact that this retrospective study spanned an extended period of time, in which we initially used the BBS in clinical practice, and later switched to the Mini-BESTest, as this test shows less ceiling effects [29]. Nevertheless, we believe that the current findings may help clinicians and therapists to better relate individual movement patterns to balance and gait capacities and, thus, to select an optimal treatment approach for individual patients with HSP, including ankle–foot surgery (in case of structural ankle–foot deformities) and balance-assistive devices, to reduce the dependence on hip strategies [4, 15].

## Declarations

### Data sharing and data accessibility

The data that support the findings of this study are available from the corresponding author upon reasonable request.
